# Safety and efficacy of pyronaridine-artesunate in uncomplicated acute malaria: an integrated analysis of individual patient data from six randomized clinical trials

**DOI:** 10.1186/1475-2875-12-70

**Published:** 2013-02-21

**Authors:** Stephan Duparc, Isabelle Borghini-Fuhrer, Carl J Craft, Sarah Arbe-Barnes, Robert M Miller, Chang-Sik Shin, Lawrence Fleckenstein

**Affiliations:** 1 Medicines for Malaria Venture (MMV), International Center Cointrin, Route de Pré-Bois 20, PO Box 1826, CH-1215, Geneva 15, Switzerland; 2 Current address: Libertyville, IL, USA; 3 Aptiv Solutions, Stevenage BioScience Catalyst, SG1 2FX, Stevenage, UK; 4 Shin Poong Pharmaceutical Co Ltd, Seoul, Republic of Korea; 5 College of Pharmacy, University of Iowa (UoI), Iowa City, IA, USA

**Keywords:** Pyronaridine-artesunate, Artemether-lumefantrine, Mefloquine + artesunate, Malaria, *Plasmodium falciparum*, *Plasmodium vivax*, Randomized clinical trial

## Abstract

**Background:**

Pyronaridine-artesunate (PA) is indicated for the treatment of acute uncomplicated *Plasmodium falciparum* and *Plasmodium vivax* malaria.

**Methods:**

Individual patient data on safety outcomes were integrated from six randomized clinical trials conducted in Africa and Asia in patients with microscopically confirmed *P. falciparum* (five studies) or *P. vivax* (one study) malaria. Efficacy against *P. falciparum* was evaluated across three Phase III clinical trials.

**Results:**

The safety population included 2,815 patients randomized to PA, 1,254 to comparators: mefloquine + artesunate (MQ + AS), artemether-lumefantrine (AL), or chloroquine. All treatments were generally well tolerated. Adverse events occurred in 57.2% (1,611/2,815) of patients with PA *versus* 51.5% (646/1,254) for comparators, most commonly (PA; comparators): headache (10.6%; 9.9%), cough (5.9%; 5.6%) and anaemia (4.5%; 2.9%). Serious averse events were uncommon for all treatments (0–0.7%). Transient increases in alanine aminotransferase and aspartate aminotransferase were observed with PA but did not lead to any clinical sequelae. For *P. falciparum* malaria, day-28 PCR-corrected adequate clinical and parasitological response with PA was 93.6% ([1,921/2,052] 95% CI 92.6, 94.7) in the intent-to-treat population and 98.5% ([1,852/1,880] 95% CI 98.0, 99.1) in the per-protocol population. Median parasite clearance time was 24.1 h with PA, 31.9 h with MQ + AS, and 24.0 h with AL. Median fever clearance time was 15.5 h with PA, 15.8 h with MQ + AS, and 14.0 h with AL. By day 42, *P. falciparum* gametocytes had declined to near zero for all treatments.

**Conclusions:**

Pyronaridine-artesunate was well tolerated with no safety concerns with the exception of mostly mild transient rises in transaminases. Efficacy was high and met the requirements for use as first-line therapy. Pyronaridine-artesunate should be considered for inclusion in malaria treatment programmes.

**Trial registration:**

Clinicaltrials.gov: NCT00331136; NCT00403260; NCT00422084; NCT00440999; NCT00541385; NCT01594931

## Background

More than three billion people are at risk of malaria [1]. Despite recent progress in malaria control, there were around 216 million episodes of clinical infection in 2010 [1]. Most malaria deaths, around 655,000 each year, occur from *Plasmodium falciparum* infection in children under five years of age [1,2]. However, the important contribution of *Plasmodium vivax* infection to global malaria morbidity is now also being recognized [3-6].

For *P. falciparum* malaria, artemisinin-based combination therapy (ACT) is generally recommended by the World Heath Organization (WHO) [7]. Parasite burden and fever are rapidly reduced with ACT and efficacy remains high in most regions [7]. Artemisinin tolerance – observed as extended parasite clearance times with ACT treatment – has emerged among *P. falciparum* from the Cambodia–Thailand border areas [8-15], with some evidence of resistance spreading at the Thailand–Myanmar border [16]. For *P. vivax*, the WHO recommends ACT in areas where chloroquine resistance has emerged [7]; most notably Indonesia, though there are also reports from further east in the Malay archipelago to Papua New Guinea and Vietnam as well as from South America and Oceania [17,18]. Using ACT in areas co-endemic for *P. falciparum* and *P. vivax* has also been suggested as a strategy to overcome difficulties in differential diagnosis and in cases of mixed infection [17,19,20].

Pyronaridine-artesunate (PA) (3:1 ratio) is a novel ACT indicated for the treatment of acute uncomplicated *P. falciparum* or *P. vivax* malaria [15,21-25]. *In vitro* studies indicate potent activity against recent isolates of multidrug-resistant strains of both *P. falciparum* and *P. vivax* from Papua, Indonesia [26], against Kenyan *P. falciparum* isolates [27], and against around half of chloroquine-resistant *P. falciparum* strains from the China–Myanmar border area [28]. The PA clinical development programme included two Phase II trials and three Phase III trials in children and adults from Africa and Asia with uncomplicated *P. falciparum* malaria [15,22,24,25], as well as one comparative Phase III trial in children and adults with uncomplicated *P. vivax* infection [23]. The programme incorporated parallel development of an adult tablet and a paediatric granule formulation [22], shown in a Phase II study to display similar bioavailability [24]. In all four Phase III studies, PA efficacy was non-inferior to the comparator anti-malarial drugs and PA was generally well tolerated [15,22,23,25].

The objective of this paper was to evaluate PA safety outcomes by integrating individual patient data from two Phase II and four Phase III randomized clinical trials [15,22-25]. Efficacy outcomes for PA in uncomplicated *P. falciparum* malaria were examined descriptively across the three Phase III trials that included patients with this infection [15,22,25].

## Methods

### Ethics statement

The clinical trials were conducted in accordance with the Declaration of Helsinki (Tokyo 2004), Good Clinical Practice and applicable regulations. Trial protocols were approved by the independent ethics committee of each study centre. All patients or their guardians provided informed written or witnessed oral consent; assent was required from children able to understand the study.

### Source data

Individual patient data were integrated from six PA randomized clinical trials, two Phase II studies and four Phase III studies, conducted in sub-Saharan Africa, Southeast Asia and India between 2005 and 2008 (Table 1) [15,22-25]. Further details of the trial protocols are supplied in the published reports [15,22-25].

**Table 1:** Summary of pyronaridine-artesunate Phase II/III randomized clinical trials included in the integrated analysis

**Protocol**	**SP-C-002-05**	**SP-C-003-05 [**[24]**]**	**SP-C-004-06 [**[15]**]**	**SP-C-005-06 [**[25]**]**	**SP-C-007-07 [**[22]**]**	**SP-C-006-06 [**[23]**]**
Phase	II	II	III	III	III	III
Region/country	SE Asia, Africa	Gabon	SE Asia, India, Africa	Africa, SE Asia	Africa, SE Asia	SE Asia, India
Year	2005–2006	2006	2007–2008	2007–2008	2007–2008	2007–2008
Design	DB/MC/DF	OL/DF	OL/MC/CM	DB/DD/MC/CM	OL/MC/CM	DB/DD/MC/CM
Pathogen	*P. falciparum*	*P. falciparum*	*P. falciparum*	*P. falciparum*	*P. falciparum*	*P. vivax*
Parasite count^a^, μL^-1^	1000–100,000	1000–200,000	1000–100,000	1000–100,000	1000–200,000	≥250
Patient age, years	15–60	2–14	3–60	3–60	≤12	3–60
Patient weight, kg	35–75	10–40	20–90	20–90	≥5– <25	20–90
PA target dose^b^, mg/kg/day	6:2, 9:3, 12:4	6:2, 9:3, 12:4 Granule: 9:3	7.2:2.4–13.8:4.6	7.2:2.4–13.8:4.6	6.7:2.2–13.3:4.4	7.2:2.4–13.8:4.6
PA dose form	Tablet	Tablet and granule	Tablet	Tablet	Granule	Tablet
Comparator	–	–	MQ + AS	AL	AL^b^	CQ

Study design: CM, comparative; DB, double-blind; DD, double-dummy; DF, dose-finding; MC, multi-centre; OL, open label.

Comparators: MQ + AS, mefloquine plus artesunate; AL, artemether-lumefantrine; CQ, chloroquine.

^a^ Asexual parasite count for *P. falciparum* and including at least 50% asexual parasites for *P. vivax.*

^b^ Crushed tablets.

Details of the studies included in this analysis are summarized in Table 1. The two Phase II studies were non-comparative; one was a dose escalation study to examine the safety and pharmacokinetics of the PA tablet and granule formulations in children with *P. falciparum* (SP-C-003-05) [24], the other was a dose ranging study (SP-C-002-05) of PA tablets in adults with *P. falciparum* malaria. Of the four Phase III studies, three were conducted in subjects with *P. falciparum*[15,22,25], two studies used PA tablets [15,25], and one the PA granule formulation [22]. The remaining Phase III study included subjects with *P. vivax* malaria [23]. All four Phase III studies were non-inferiority studies against comparators recommended as first-line anti-malarial therapy in the relevant countries. The non-inferiority margin was 5% in the two *P. falciparum* tablet studies that included both children and adults (SP-C-004-06, SP-C-005-06), and 10% in the paediatric granule *P. falciparum* study (SP-C-007-07) and the *P. vivax* study (SP-C-006-06).

### Treatments

Pyronaridine-artesunate (Shin Poong Pharmaceutical Company Ltd, Ansan, South Korea) was supplied as tablets (180:60 mg per tablet) or as a paediatric granule formulation presented in sachets (60:20 mg per sachet). In the Phase III studies, PA was given once daily for three days (day 0, 1, 2), dosed by body weight as follows, for tablets: 20–25 kg one tablet, 26–44 kg two tablets, 45–64 kg three tablets, 65–90 kg four tablets, i e, 7.2:2.4 to 13.8:4.6 mg/kg/dose; and for granules, ≥5– <9 kg one sachet, 9– <17 kg two sachets, 17– <25 kg three sachets, i e, 6.7:2.2 to 13.3:4.4 mg/kg/dose.

The comparators in the PA Phase III *P. falciparum* studies were dosed by body weight. Artemether-lumefantrine (AL) was given twice daily for three days, at 0.9:5.3 to 2.4:14.4 mg/kg/dose for tablets in SP-C-005-06 and 1.3:8.0 to 4.0:24.0 mg/kg/dose for crushed tablets in SP-C-007-07. Dosing of AL was with or without food based on local practices. Mefloquine + artesunate (MQ + AS) was given as a loose combination, once daily for three days at 5.6:2.2 to 12.5:5.0 mg/kg/dose in study SP-C-004-06. In the Phase III *P. vivax* study (SP-C-006-06), the comparator chloroquine (CQ) was given once daily for three days at a daily dose in children of 10 mg/kg on days 0 and 1 and 5 mg/kg on day 2 and for adults of 620 mg on days 0 and 1 and 310 mg on day 2.

### Patients and procedures

Patients and procedures are described in detail elsewhere [15,22-25]. In brief, patients of either gender were eligible if they had *P. falciparum* mono-infection, or in SP-C-006-06 *P. vivax* mono-infection, in all cases microscopically confirmed within defined limits (Table 1), with fever or a history of fever while meeting age/body weight criteria for each study (Table 1). Patients were excluded if they had significant co-morbid illness, recent anti-malarial therapy, known hypersensitivity to study drugs, evidence of severe malnutrition, if they were pregnant (test required) or breastfeeding, or had participated previously in a PA clinical trial.

At screening, a medical history was taken and a physical examination performed. All enrolled patients were hospitalized from day 0 to 3. Follow-up assessments were at days 7, 14, 21, 28, 35 and 42. Vital signs and malaria signs/symptoms were monitored throughout the study. Temperature was taken at screening, every 8 h over ≥72 h following the first dose or until two normal readings between 7 and 25 h apart, then at each visit or as clinically indicated. Venous blood samples were taken for clinical biochemistry, haematology and urinalysis testing and 12-lead electrocardiographs were performed at screening and then at intervals as specified in the protocol.

Parasitological assessments were performed according to WHO guidelines [29]. Giemsa-stained thick blood slides were prepared before each dose, every 8 h (±1 h) following first dose administration for ≥72 h or until parasite clearance (two consecutive negative readings 7 to 25 h apart), and at subsequent visits. For *P. falciparum*, asexual and gametocyte parasite counts were performed separately; for *P. vivax* asexual and gametocyte parasite counts were recorded separately at screening and total parasite counts recorded at all other assessments. Thin blood slides were prepared for parasite identification at screening and from day 7 at all follow-up visits. In the *P. falciparum* Phase III studies, recrudescence was determined by PCR genotyping using *P. falciparum* genes *msp* 1, *msp* 2, and *glurp*; defined as at least one matching allelic band in all markers between baseline and post-day-7 samples [30,31].

### Outcomes

Safety outcomes were adverse event incidence and severity (categorized using MedDRA version 10.1), laboratory abnormalities (graded according to the Division of Microbiology and Infectious Diseases Toxicity Scale), and electrocardiograph abnormalities.

Efficacy outcomes were integrated for the Phase III *P. falciparum* studies only and were defined according to WHO methods [29]. The primary efficacy outcome was day-28 PCR-corrected adequate clinical and parasitological response (ACPR), defined as clearance of asexual parasitaemia without recrudescence or previous treatment failure. Day-28 uncorrected ACPR was a secondary endpoint.

Secondary efficacy endpoints were parasite clearance time (time from first dose to asexual aparasitaemia for two consecutive negative readings 7–25 h apart); fever clearance time (time from first dose to apyrexia for two consecutive normal temperature readings 7–25 h apart); and the proportion of subjects aparasitic and proportion apyretic at day 1 (24 h after the first dose), day 2 (48 h after the first dose), and day 3 (72 h after the first dose). The proportion of patients with gametocytes at each assessment was an exploratory endpoint.

### Statistical methods

The safety analysis included data from all Phase II and Phase III studies and was conducted on the safety population, i e, all patients who had received any amount of study drug. Descriptive statistics were provided for safety outcomes.

The efficacy analysis included data from the Phase III *P. falciparum* trials and in the integrated analysis was conducted primarily on the intent-to-treat (ITT) population, i e, all randomized patients with *P. falciparum* malaria who received any amount of study medication. This definition was consistent across all the Phase III *P. falciparum* studies. Descriptive statistics were provided for efficacy outcomes.

Descriptive sub-group analyses were performed for day-28 PCR-corrected ACPR for the ITT population as follows: region (Asia, Africa); age (<5, 5–12, >12– <18, ≥18 years); gender (male, female); weight (<17, ≥17– <25, ≥25 kg); previous malaria episode (yes, no); number of previous malaria episodes in the last 12 months (0, 1, >1); baseline parasitaemia (≤5,000, >5,000–10,000, >10,000 μL^-1^); PA formulation (granule, tablet); and PA actual dose (≤8.5:2.8, >8.5:2.8–9.5:3.2, >9.5:3.2–11.0:3.7, >11.0:3.7 mg/kg/day).

Post-hoc analyses of time to recrudescence and time to re-infection conducted using Kaplan-Meier estimates (log-rank test) were conducted for the integrated analysis; subjects without the event were censored at the last available parasite assessment date.

Kaplan-Meier estimates were used to evaluate parasite clearance time and fever clearance time. Subjects without parasite or fever clearance within 72 h after the first treatment dose were censored at that time point. An additional analysis of parasite clearance time in Cambodia (Pailin at the Cambodia–Thailand border) *versus* Thailand (Mae Sot and Mae Ramat) was performed without censoring at 72 h. The proportion of subjects with parasite clearance and the proportion of subjects with fever clearance on days 1, 2 and 3 were calculated using Kaplan-Meier estimates. Log_10_ area under the curve (AUC) for gametocyte density was calculated in count · day/μL based on a log_10_ transformation. Statistical analysis was performed using SAS (Version 9.1).

## Results

### Patients

Overall, 2,817 patients were randomized to PA and 1,254 to comparators (MQ + AS, AL and CQ). Two subjects did not receive PA and were excluded from the safety population (*n* = 2,815) (Table 2). A similar proportion of patients in the PA group (14.4% [405/2,815]) discontinued treatment as for comparators (17.6% [221/1,254]). In the efficacy (ITT) population, 2,052 subjects with *P. falciparum* malaria were treated with PA and 1,026 with comparators (MQ + AS or AL) (Table 2). Subject disposition for the efficacy ITT population was similar to that of the safety population (Table 2).

**Table 2:** Subject disposition

**Subjects**	**PA safety**	**PA efficacy (ITT)**	**MQ + AS safety/efficacy**	**AL safety/efficacy**	**CQ safety**	**All comparators safety**^**a**^
Treated^b^	2815	2052	423	603	228	1254
Completed treatment^c^	2773 (98.5)	2017 (98.3)	418 (98.8)	587 (97.3)	220 (96.5)	1225 (97.7)
Completed study	2410 (85.6)	1748 (85.2)	357 (84.4)	489 (81.1)	187 (82.0)	1033 (82.4)
Discontinued:	405 (14.4)	304 (14.8)	66 (15.6)	114 (18.9)	41 (18.0)	221 (17.6)
Adverse event	46 (1.6)	32 (1.6)	4 (0.9)	10 (1.7)	2 (0.9)	16 (1.3)
Consent withdrawn	26 (0.9)	19 (0.9)	6 (1.4)	11 (1.8)	5 (2.2)	22 (1.8)
Insufficient therapeutic effect	11 (0.4)	0	0	0	0	0
Lost to follow-up	99 (3.5)	69 (3.4)	25 (5.9)	10 (1.7)	12 (5.3)	47 (3.7)
Protocol violation	9 (0.3)	4 (0.2)	0	1 (0.2)	0	1 (0.1)
Malaria infection	186 (6.6)	174 (8.5)	29 (6.9)	78 (12.9)	21 (9.2)	128 (10.2)
Other	28 (1.0)	6 (0.3)	2 (0.5)	4 (0.7)	1 (0.4)	7 (0.6)

Data are n (%).

PA, pyronaridine-artesunate; MQ + AS, mefloquine plus artesunate; AL, artemether-lumefantrine; CQ, chloroquine.

^a^ For comparators, MQ + AS, AL and CQ were included in the safety analysis; MQ + AS and AL in the efficacy analysis.

^b^ Received at least one dose of study medication.

^c^ Received all planned doses of study medication.

In the PA group (safety population), 91.9% (2,587/2,815) of patients were treated for *P. falciparum* and 8.1% (228/2,815) for *P. vivax* malaria, 49.3% (1,389/2,815) of subjects were from Asia, 50.7% (1,426/2,815) from Africa and mean patient age was 20.6 years (Table 3). Overall in the safety population, the PA group and the combined comparators group were well balanced for baseline demographic and clinical characteristics (Table 3). However, for individual comparators, integration of the data led to imbalances in patient age and the regions from which patients were recruited because of the different study entry criteria (Table 3).

**Table 3:** Patient baseline characteristics

**Characteristic**	**PA safety**^**a**^	**PA efficacy (ITT)**^**a**^	**MQ + AS safety/efficacy**	**AL safety/efficacy**	**CQ safety**	**All comparators safety**^**a**^
	***n*** **= 2815**	***n*** **= 2052**	***n*** **= 423**	***n*** **= 603**	***n *=228**	***n*** **= 1254**
Female gender, n (%)	956 (34.0)	751 (36.6)	93 (22.0)	288 (47.8)	64 (28.1)	445 (35.5)
Mean age, years (SD) [range]	20.6 (12.9) [0.3–60.4]	18.6 (12.6) [0.3–60.4]	25.6 (12.5) [5.5–59.7]	14.6 (11.3) [0.3–57.0]	26.6 (10.9) [7.0–58.0]	20.5 (12.9) [0.3–59.7]
Age category, years, n (%)						
≤1 year	9 (0.3)	9 (0.4)	0	3 (0.5)	0	3 (0.2)
>1– <5	173 (6.1)	150 (7.3)	0	68 (11.3)	0	68 (5.4)
5–12	704 (25.0)	656 (32.0)	61 (14.4)	274 (45.4)	9 (3.9)	344 (27.4)
>12– <18	401 (14.2)	316 (15.4)	53 (12.5)	92 (15.3)	28 (12.3)	173 (13.8)
≥18	1528 (54.3)	921 (44.9)	309 (73.0)	166 (27.5)	191 (83.8)	666 (53.1)
Region, n (%)^b^						
Africa	1426 (50.7)	1223 (59.6)	79 (18.7)	531 (88.1)	0	610 (48.6)
Asia	1389 (49.3)	829 (40.4)	344 (81.3)	72 (11.9)	228 (100)	644 (51.4)
Mean body weight, kg (SD) [range]	42.1 (16.5) [6.0–89.5]	39.5 (17.1) [6.0–89.5]	47.3 (12.1) [20.0–75.0]	34.6 (18.1) [7.2–86.0]	49.4 (10.2) [20.0–80.0]	41.6 (16.5) [7.2–86.0]
Malaria in last 12 months, n (%)	*n* = 2551	*n* = 2045	*n* = 423	*n* = 600	*n* = 228	*n* = 1251
0	1431 (56.1)	1064 (52.0)	258 (61.0)	269 (44.8)	123 (53.9)	650 (52.0)
1	498 (19.5)	444 (21.7)	92 (21.7)	116 (19.3)	58 (25.4)	266 (21.3)
>1	622 (24.4)	537 (26.3)	73 (17.3)	215 (35.8)	47 (20.6)	335 (26.8)
*P. falciparum* parasitaemia, μL^-1^, n (%)^c^						
≤5000	691 (26.8)	521 (25.4)	113 (26.7)	161 (26.7)	–	274 (26.7)
>5000–10,000	394 (15.3)	293 (14.3)	72 (17.0)	81 (13.5)	–	153 (14.9)
>10,000	1497 (58.0)	1238 (60.3)	238 (56.3)	360 (59.8)	–	598 (58.3)
*P. vivax* geometric mean asexual parasitaemia, μL^–1^ [range]	6914.8 [466–92500]	–	–	–	6145.8 [366–77035]	–
Mean haemoglobin, g/dL (SD)	11.9 (2.0)	11.8 (2.0)	12.1 (2.0)	11.5 (1.9)	12.3 (1.8)	11.9 (1.9)

PA, pyronaridine-artesunate; MQ + AS, mefloquine plus artesunate; AL, artemether-lumefantrine; CQ, chloroquine.

^a^ The safety analysis included data from all Phase II and Phase III studies. The efficacy analysis included data from the Phase III *P. falciparum* trials. For comparators, MQ + AS, AL and CQ were included in the safety analysis; MQ + AS and AL in the efficacy analysis.

^b^ All subjects from African centres were of Black ethnicity except one in the AL group who was Asian/Oriental; all patients from Asian centres were of Asian/Oriental ethnicity.

^c^ Baseline *P. falciparum* parasite count was not available for five patients in the PA group and one patient in the AL group.

### Safety

In the safety population, medication was taken as planned for 98.9% (2,785/2,815) of patients receiving PA and 98.6% (1,236/1,254) of those receiving comparators. All drug treatments were generally well tolerated. The number of patients with at least one treatment-emergent adverse event was similar for PA (57.2% [1,611/2,815]) and comparators (51.5% [646/1,254]), as was the number of patients thought to have adverse events related to drug therapy: 25.2% (708/2,815) and 25.5% (320/1,254), respectively. The nature and incidence of adverse events due to any cause was similar for PA *versus* combined comparators (Table 4). The most common adverse events were headache (10.6% PA; 9.9% comparators), cough (5.9% PA; 5.6% comparators) and anaemia (4.5% PA; 2.9% comparators) (Table 4).

**Table 4:** **Treatment-emergent adverse events of any cause occurring in ≥2**% **of all subjects in any treatment group (safety population)**

**Adverse event**	**PA**	**MQ + AS**	**AL**	**CQ**	**All comparators**
	**(*n*** **= 2815)**	**(*n*** **= 423)**	**(*n*** **= 603)**	**(*n*** **= 228)**	**(*n*** **= 1254)**
Any adverse event	1611 (57.2)	190 (44.9)	384 (63.7)	72 (31.6)	646 (51.5)
**Blood and lymphatic system disorders**					
Anaemia	128 (4.5)	17 (4.0)	18 (3.0)	1 (0.4)	36 (2.9)
Basophilia	9 (0.3)	9 (2.1)	1 (0.2)	0	10 (0.8)
Eosinophilia	101 (3.6)	5 (1.2)	27 (4.5)	4 (1.8)	36 (2.9)
Lymphocytosis	21 (0.7)	0	16 (2.7)	0	16 (1.3)
Neutropenia	55 (2.0)	0	27 (4.5)	0	27 (2.2)
**Gastrointestinal disorders**					
Abdominal pain	98 (3.5)	7 (1.7)	31 (5.1)	2 (0.9)	40 (3.2)
Diarrhoea	41 (1.5)	9 (2.1)	6 (1.0)	2 (0.9)	17 (1.4)
Vomiting	124 (4.4)	9 (2.1)	23 (3.8)	7 (3.1)	39 (3.1)
**General disorders**					
Fatigue	49 (1.7)	6 (1.4)	3 (0.5)	11 (4.8)	20 (1.6)
Influenza-like illness	49 (1.7)	0	21 (3.5)	0	21 (1.7)
Pyrexia	66 (2.3)	4 (0.9)	18 (3.0)	2 (0.9)	24 (1.9)
**Infections and infestations**					
Bronchitis	52 (1.8)	6 (1.4)	12 (2.0)	3 (1.3)	21 (1.7)
Nasopharyngitis	74 (2.6)	11 (2.6)	8 (1.3)	6 (2.6)	25 (2.0)
Rhinitis	19 (0.7)	2 (0.5)	12 (2.0)	0	14 (1.1)
Upper RTI	100 (3.6)	4 (0.9)	31 (5.1)	0	35 (2.8)
Urinary tract infection	34 (1.2)	3 (0.7)	12 (2.0)	1 (0.4)	16 (1.3)
**Investigations**					
AST increased	63 (2.2)	1 (0.2)	14 (2.3)	0	15 (1.2)
Blood albumin decreased	21 (0.7)	0	16 (2.7)	0	16 (1.3)
Blood CPK increased	26 (0.9)	4 (0.9)	3 (0.5)	8 (3.5)	15 (1.2)
Blood glucose decreased	33 (1.2)	0	19 (3.2)	0	19 (1.5)
ECG QT prolongation	6 (0.2)	5 (1.2)	1 (0.2)	6 (2.6)	12 (1.0)
Eosinophil count increased	58 (2.1)	6 (1.4)	2 (0.3)	0	8 (0.6)
Platelet count increased	59 (2.1)	3 (0.7)	20 (3.3)	0	23 (1.8)
**Metabolism and nutrition disorders**					
Anorexia	85 (3.0)	13 (3.1)	10 (1.7)	10 (4.4)	33 (2.6)
**Musculoskeletal and connective tissue disorders**					
Myalgia	107 (3.8)	19 (4.5)	1 (0.2)	21 (9.2)	41 (3.3)
**Nervous system disorders**					
Dizziness	39 (1.4)	28 (6.6)	1 (0.2)	6 (2.6)	35 (2.8)
Headache	297 (10.6)	44 (10.4)	46 (7.6)	34 (14.9)	124 (9.9)
**Respiratory, thoracic and mediastinal disorders**					
Cough	167 (5.9)	10 (2.4)	55 (9.1)	5 (2.2)	70 (5.6)

Data are n (%). PA, pyronaridine-artesunate; MQ + AS, mefloquine plus artesunate; AL, artemether-lumefantrine; CQ, chloroquine; RTI, respiratory tract infection; AST aspartate aminotransferase; CPK, creatine phosphokinase; ECG, electrocardiograph.

Values underlined are for adverse events occurring in ≥2% of patients in one group *versus* any other group.

Serious adverse events occurred in 0.6% (18/2,815) of patients in the PA group and in 0.4% (5/1,254) in the combined comparators. There were no deaths. One patient in the PA group had two serious adverse events that were considered treatment-related (hepatic enzyme increased and abortion incomplete) and one patient receiving MQ + AS had two treatment-related serious adverse events (convulsion and grand mal convulsion). All other serious adverse events were considered not to be related to treatment: in the PA group there were two cases each of pyrexia, malaria, typhoid fever and urinary tract infection, and one case each of anaemia, cardiac failure, abdominal pain, limb abscess, cholera, paronychia, parotitis, pneumonia, acute pyelonephritis, wound infection, abortion complete and depression; for comparators there were two cases of cerebral malaria and one of immunosuppression.

Overall, adverse events leading to study withdrawal occurred in 1.6% (44/2,815) of patients in the PA group and 1.0% (13/1,254) in the comparator group; vomiting was the reason in 1.1% (32/2,815) and 0.9% (11/1,254) of cases, respectively. In the PA group, other reasons for study withdrawal were *P. falciparum* infection (0.3%; 9/2,815), malaria (0.1%; 3/2,815) and one case each of nausea and fatigue. In the comparator group there were two cases of cerebral malaria (0.2%) leading to study withdrawal. Of the patients who were withdrawn from the study because of an adverse event, drug treatment was discontinued in 2.2% (27/1,254) of patients in the PA group (26 instances of vomiting, one of nausea, one of malaria) and in 1.0% (12/1,254) of patients receiving comparators (10 vomiting, two cerebral malaria).

**Figure 1:**
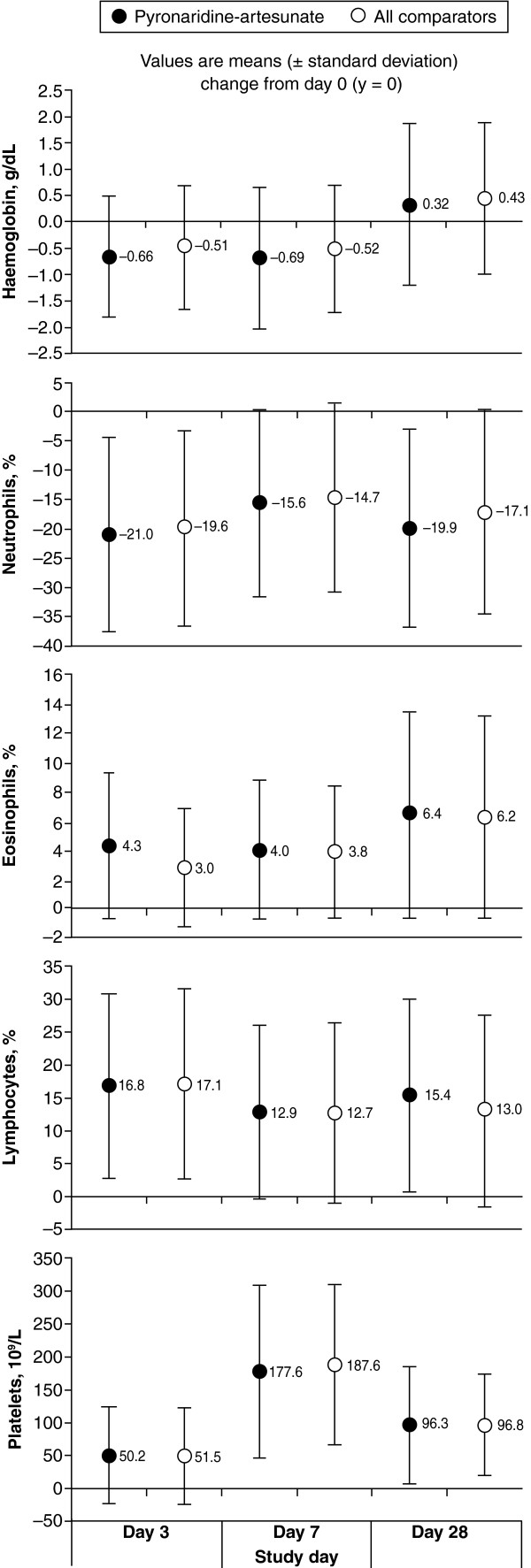
**Changes from baseline in haematology parameters in patients receiving pyronaridine-artesunate or combined comparators (safety population).** NB: Day-28 values are available for the *Plasmodium vivax* study only [23].

There were no major differences between PA and comparators in haematology parameters over the course of the study (Figure 1). For all treatment groups, there was a small decrease in haemoglobin concentration on day 3 (mean <1 g/dL) with recovery to baseline levels by day 28, and corresponding changes in haematocrit and red blood cells. Proportional decreases in neutrophils from baseline were associated with mean proportional increases in lymphocytes and eosinophils. Platelet counts were low at baseline and had increased by day 7 in all treatment groups. There were no clinically meaningful changes from baseline for white blood cells, monocytes or basophils in any treatment group.

**Figure 2:**
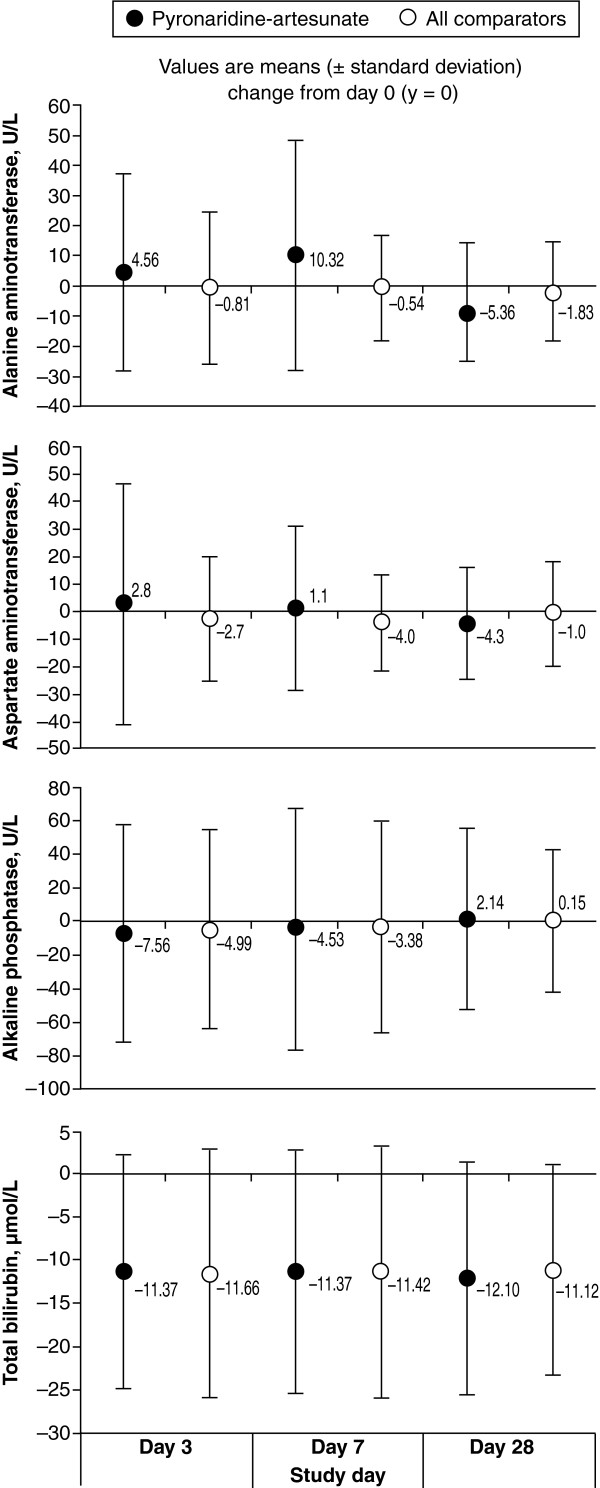
**Changes from baseline in liver enzymes and total bilirubin in patients receiving pyronaridine-artesunate or combined comparators (safety population).** NB: Day-28 values are available for the *Plasmodium vivax* study only [23].

No clinically important changes from baseline were observed in total bilirubin, alkaline phosphatase, creatinine, albumin, creatine kinase, urea, sodium, potassium, glucose or blood urea nitrogen in any treatment group. In the PA group, both alanine aminotransferase (ALT) and aspartate aminotransferase (AST) were increased on day 3 and day 7 *versus* baseline, and normalized by day 28 (Figure 2). In the PA group, treatment-emergent Grade 3/4 toxicities for ALT were recorded for 0.4% (11/2,750) of patients on day 3 and 0.9% (24/2,709) on day 7; for AST the incidence was 0.5% (13/2,757) and 0.3% (9/2,711), respectively. For comparators, in the MQ + AS group 0.2% (1/404) of patients had an ALT Grade 3 toxicity on day 7. In the AL group, 0.2% (1/582) of patients had an ALT Grade 4 toxicity on day 3, and for AST 0.5% (3/583) had Grade 3 toxicity on day 3 and 0.2% (1/581) on day 7. Only one patient in the PA group had a serious adverse event related to a Grade 3/4 biochemistry value, hepatic enzyme increased: Grade 3 AST and ALT values on day 7 (295 U/L and 272 U/L, respectively), decreasing to near normal levels by day 28 (54 U/L, 25 U/L, respectively); total bilirubin was normal at all assessments; ALP was normal except for a Grade 1 toxicity on day 7 (168 U/L). The proportion of patients with ALT and/or AST >3x the upper limit of normal (ULN) plus peak total bilirubin >2xULN in the PA group was 0.2% (7/2,815) *versus* 0.3% (2/603) for AL; one of these patients receiving PA also had a clinically important increase in ALP. There were no cases in the MQ + AS or CQ groups. No clinically important changes were recorded for urine glucose, protein and blood screening.

The incidence of treatment-emergent clinically important abnormal electrocardiograph results was 1.1% (30/2,752) for PA, 0.7% (3/417) for MQ + AS, 0.3% (2/588) for AL and 2.7% (6/222) for CQ. An adverse event of QT interval prolongation was recorded for 0.2% (6/2,815) of patients receiving PA, 1.2% (5/423) receiving MQ + AS, 0.2% (1/603) receiving AL and 2.6% (6/228) receiving CQ. The proportion of patients with an adverse event of bradycardia was 1.1% (31/2,815) for PA and 0.8% (5/603) for AL with no cases in the MQ + AS or CQ groups. No electrocardiograph abnormality required drug discontinuation or withdrawal from the study.

### Efficacy

**Figure 3:**
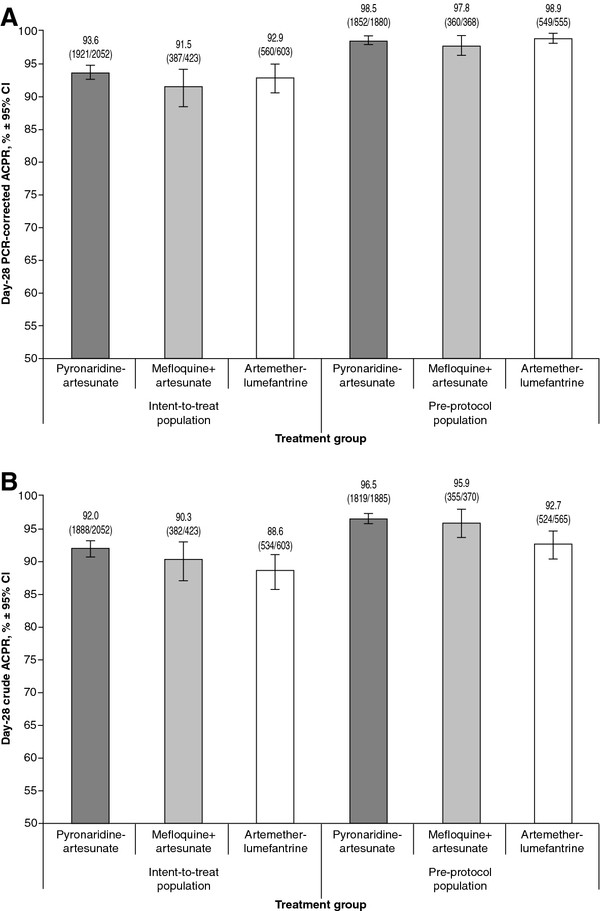
**Day-28 adequate clinical and parasitological response (ACPR): A) PCR-corrected; B) uncorrected.** Two-sided 95% confidence intervals (Wald) were adjusted for study.

Efficacy was evaluated in an integrated analysis of the Phase III *P. falciparum* studies SP-C-004-06 [15] SP-C-005-06 [25] and SP-C-007-07 [22]. Day-28 PCR-corrected ACPR for PA was 93.6% ([1,921/2,052] 95% CI 92.6, 94.7) in the ITT population and 98.5% ([1,852/1,880] 95% CI 98.0, 99.1) in the PP population; similar to comparators (Figure 3A). For day-28 uncorrected ACPR, there was no difference between PA and comparators in the ITT or PP populations (Figure 3B).

Sub-group analysis provided similar day-28 PCR-corrected ACPR rates (ITT population) regardless of region, age group, gender, weight, previous malaria, number of episodes of malaria in the last 12 months, or baseline parasitaemia (Table 5). There was no difference in day-28 PCR-corrected ACPR (ITT population) between the PA granule and tablet formulations: 93.8% ([333/355] 95% CI 91.3, 96.3) and 93.6% ([1,588/1,697] 95% CI 92.4, 94.7), respectively. Efficacy was similar across the actual PA dose groups: 92.2% ([365/396] 95% CI 89.5, 94.8) for ≤8.5:2.8 mg/kg/day; 93.9% ([512/545] 95% CI 91.9, 96.0) for >8.5:2.8–9.5:3.2 mg/kg/day; 94.0% ([616/655] 95% CI 92.2, 95.9) for >9.5:3.2–11.0:3.7 mg/kg/day; and 93.9% ([428/456] 95% CI 91.6, 96.1) for >11.0:3.7 mg/kg/day.

**Table 5:** **Descriptive sub-group analysis of day-28 PCR-corrected ACPR for *Plasmodium falciparum *(intent-to-treat population)**

**Sub-group**	**PA**		**MQ + AS**		**AL**	
	**(*n*** **= 2052)**		**(*n*** **= 423)**		**(*n*** **= 603)**	
	**n/N**	**% (95% CI)**	**n/N**	**% (95% CI)**	**n/N**	**% (95% CI)**
**Region**						
Asia	760/829	91.7 (89.8, 93.6)	310/344	90.1 (86.9, 93.3)	64/72	88.9 (81.5, 96.3)
Africa	1161/1223	94.9 (93.7, 96.2)	77/79	97.5 (93.9, 100)	496/531	93.4 (91.3, 95.5)
**Age group, years**						
<5	145/159	91.2 (86.7, 95.6)	–	–	63/71	88.7 (81.2, 96.3)
5–12	621/656	94.7 (92.9, 96.4)	60/61	98.4 (95.1, 100)	255/274	93.1 (90.0, 96.1)
>12– <18	297/316	94.0 (91.4, 96.6)	52/53	98.1 (94.3, 100)	86/92	93.5 (88.3, 98.6)
≥18	858/921	93.2 (91.5, 94.8)	275/309	89.0 (85.5, 92.5)	156/166	94.0 (90.3, 97.6)
**Gender**						
Male	1213/1301	93.2 (91.9, 94.6)	299/330	90.6 (87.4, 93.8)	293/315	93.0 (90.2, 95.8)
Female	708/751	94.3 (92.6, 95.9)	88/93	94.6 (90.0, 99.3)	267/288	92.7 (89.7, 95.7)
**Weight, kg**						
<17	170/188	90.4 (86.2, 94.7)	–	–	84/94	89.4 (83.0, 95.7)
17– <25	385/401	96.0 (94.1, 97.9)	38/39	97.4 (92.2, 100)	159/171	93.0 (89.1, 96.8)
≥25	1366/1463	93.4 (92.1, 94.6)	349/384	90.9 (90.9, 93.8)	317/338	93.8 (91.2, 96.4)
**Previous malaria**						
No	828/894	92.6 (90.9, 94.3)	199/220	90.5 (86.5, 94.4)	187/206	90.8 (86.8, 94.8)
Yes	1093/1157	94.5 (93.1, 95.8)	188/203	92.6 (89.0, 96.2)	373/397	94.0 (91.6, 96.3)
**Malaria in last 12 months**						
None	976/1064	91.7 (90.1, 93.4)	232/258	89.9 (86.2, 93.6)	243/269	90.3 (86.8, 93.9)
1 episode	420/444	94.6 (92.5, 96.7)	84/92	91.3 (85.4, 97.2)	111/116	95.7 (92.0, 99.4)
>1 episode	519/537	96.6 (95.1, 98.2)	71/73	97.3 (93.4, 100)	203/215	94.4 (91.3, 97.5)
**Baseline parasitaemia, μL**^**-1**^						
≤5000	487/521	93.5 (91.3, 95.6)	102/113	90.3 (84.7, 95.8)	150/161	93.2 (89.2, 97.1)
>5000–10,000	275/293	93.9 (91.1, 96.6)	64/72	88.9 (81.5, 96.3)	78/81	96.3 (92.1, 100)
>10,000	1159/1238	93.6 (92.3, 95.0)	221/238	92.9 (89.6, 96.2)	332/360	92.2 (89.4, 95.0)

–, no data available. PA, pyronaridine-artesunate; MQ + AS, mefloquine plus artesunate; AL, artemether-lumefantrine.

Two-sided confidence intervals (Wald) were adjusted for study.

**Figure 4:**
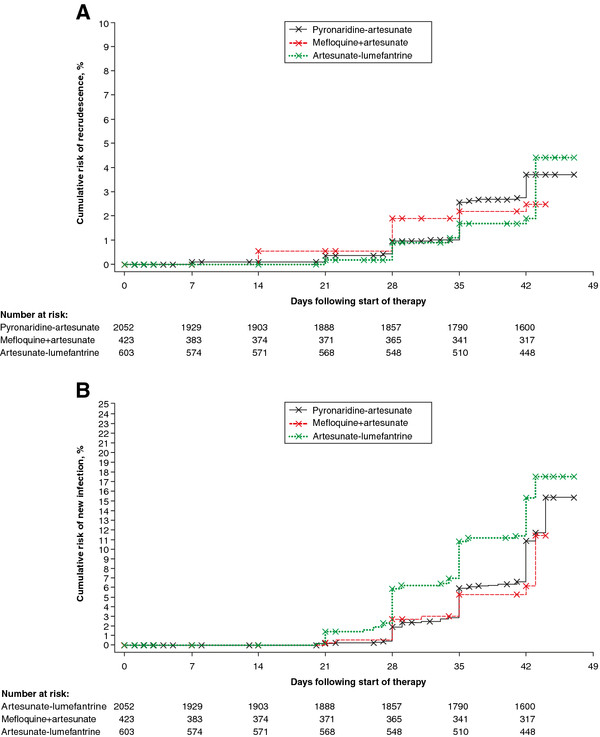
***Plasmodium falciparum *A) recrudescence rate B) re-infection rate.** Kaplan-Meier survival plot for the integrated efficacy analysis (intent-to-treat population).

Kaplan-Meier estimates of *P. falciparum* recrudescence rate and re-infection rate are shown in Figure 4. Both the recrudescence rate (Figure 4A) and the re-infection rate (Figure 4B) with PA were intermediate between MQ + AS and AL.

**Figure 5:**
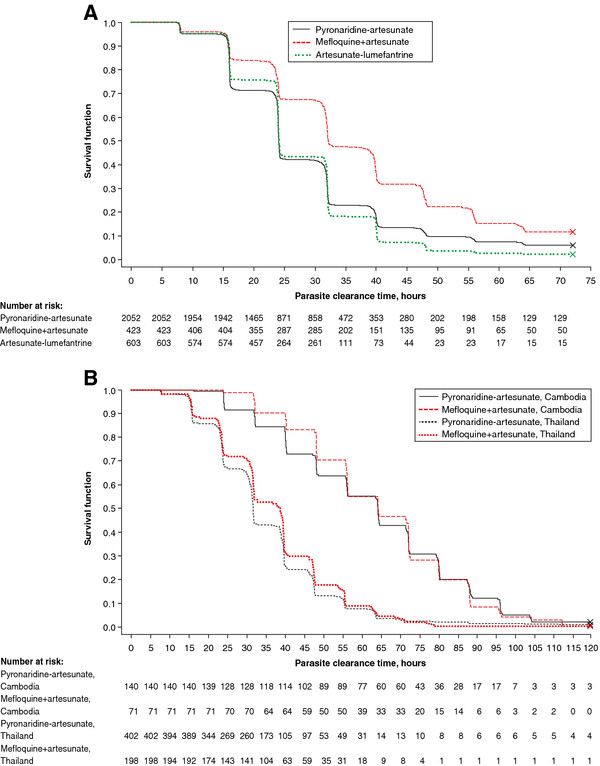
**Parasite clearance time for A) all centres; B) Cambodia *versus *Thailand.** Kaplan-Meier survival plot for the integrated efficacy analysis (intent-to-treat population).

The majority of patients in all treatment groups had *P. falciparum* clearance by day 2 (Table 6). Median parasite clearance time for *P. falciparum* (ITT population) was 24.1 h with PA, 31.9 h with MQ + AS, and 24.0 h with AL (Table 6; Figure 5A). Median parasite clearance time was extended for all treatments in Asian *versus* African centres (Table 6). In particular, median parasite clearance time was extended in Cambodia for both treatment groups at 64.1 h for PA and 64.2 h for MQ + AS (Figure 5B). In comparison, median parasite clearance time in Thailand was 31.7 h for PA and 38.7 h for MQ + AS (Figure 5B). Day-28 PCR-corrected ACPR (ITT population) in the PA group in Cambodia was 89.3% (125/140) 95% CI 82.9, 93.9 and in Thailand was 93.3% (375/402) 95% CI 90.4, 95.5. For MQ + AS group, day-28 PCR-corrected ACPR (ITT population) was 93.0% (66/71) 95% CI 84.3, 97.7 in Cambodia and 88.9% (176/198) 95% CI 83.7, 92.9 in Thailand.

**Table 6:** ***Plasmodium falciparum *parasite and fever clearance times and proportion of patients with clearance on days 1, 2, and 3 (intent-to-treat population)**

**Outcome**	**PA**	**MQ + AS**	**AL**
**Parasite clearance**			
Patients with clearance,% (n/N)	93.7 (1923/2052)	88.2 (373/423)	97.5 (588/603)
Median time to clearance, h	24.1	31.9	24.0
Africa	23.9	23.9	24.0
Asia	32.0	39.2	31.5
Day 1 clearance,% (95% CI)	48.2 (46.2, 50.2)	33.7 (30.8, 36.8)	51.5 (48.5, 54.6)
Day 2 clearance,% (95% CI)	89.9 (88.6, 91.1)	76.1 (72.5, 79.6)	92.0 (90.1, 93.6)
Day 3 clearance,% (95% CI)	94.8 (93.9, 95.6)	84.2 (81.0, 87.1)	96.1 (94.9, 97.2)
**Fever clearance**			
Patients with clearance,% (n/N)	98.0 (1589/1621)	97.8 (348/356)	97.2 (450/463)
Median time to clearance, h	15.5	15.8	14.0
Africa	8.0	8.0	8.0
Asia	16.0	16.0	16.0
Day 1 clearance,% (95% CI)	81.9 (80.1, 83.6)	74.7 (70.8, 78.4)	84.1 (81.2, 86.8)
Day 2 clearance,% (95% CI)	95.8 (94.9, 96.6)	92.2 (89.8, 94.3)	96.7 (95.4, 97.8)
Day 3 clearance,% (95% CI)	97.8 (97.1, 98.3)	95.3 (93.4, 96.8)	98.3 (97.5, 99.0)

PA, pyronaridine-artesunate; MQ + AS, mefloquine plus artesunate; AL, artemether-lumefantrine; NA, not available.

Percentages are based on available observations.

Median percentiles as well as confidence intervals and estimates of survival rates at Day 1, 2 and 3 were adjusted for study.

**Figure 6:**
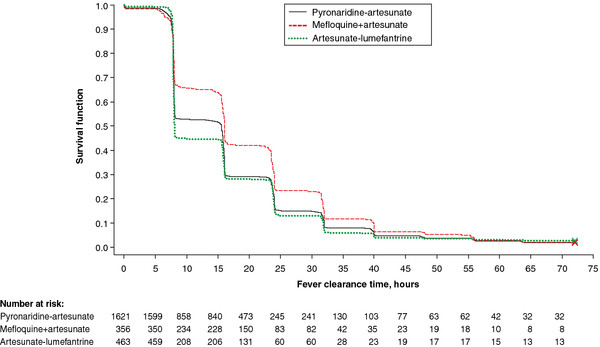
**Fever clearance time.** Kaplan-Meier survival plot for the integrated efficacy analysis (intent-to-treat population)

The majority of patients with *P. falciparum* had fever clearance by day 1 (Table 6). Median fever clearance time for *P. falciparum* in the ITT population was 15.5 h for PA, 15.8 h for MQ + AS and 14.0 h with AL (Table 6, Figure 6). Fever clearance times were extended in Asia *versus* Africa for all treatments (Table 6).

**Figure 7:**
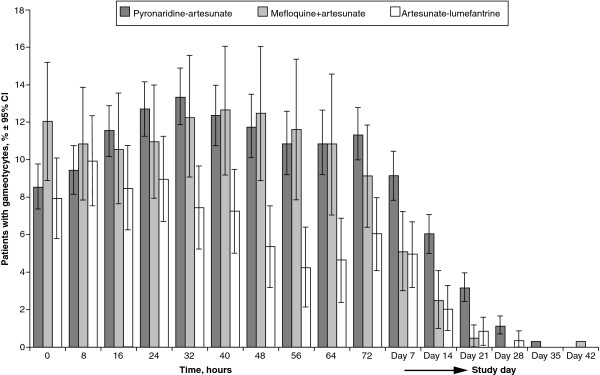
**Proportion of patients with *Plasmodium falciparum *gametocytes.** Integrated efficacy analysis (intent-to-treat population). Two-sided 95% confidence intervals (Wald) were adjusted for study.

*Plasmodium falciparum* gametocytes were present in 8.9% (276/3,078) of patients at baseline, and gradually decreased to 0% or near 0% in all treatment groups by the end of the study (Figure 7).

**Figure 8:**
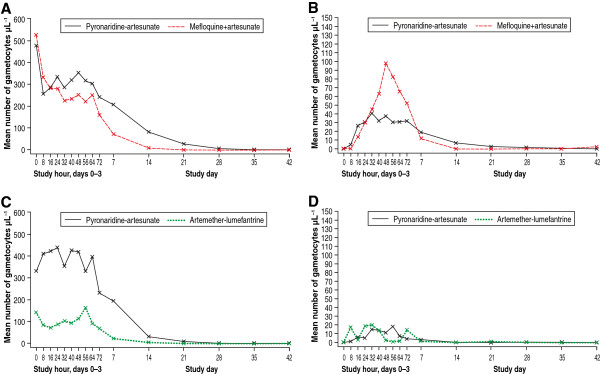
***Plasmodium falciparum *gametocyte density over time. A)** Study SP-C-004-06 comparing pyronaridine-artesunate to mefloquine + artesunate gametocyte density over time in patients with baseline gametocytes and **B)** no gametocytes at baseline and post-baseline gametocytes. **C)** Study SP-C-005-06 comparing pyronaridine-artesunate to artemether-lumefantrine gametocyte density over time in patients with baseline gametocytes and **D)** no gametocytes at baseline and post-baseline gametocytes. All data are for the intent-to-treat population.

To further investigate *P. falciparum* gametocyte carriage, data for the two studies that recruited both adults and children (SP-C-004-06 and SP-C-005-06) were reanalysed to calculate the log_10_ AUC for gametocyte density (Figure 8). In study SP-C-004-06, in patients who had gametocytes at baseline, median log_10_ [AUC gametocyte density] was 3.1 (range 0.1 to 4.4) in the PA group (n = 81) and 2.5 (range −0.2 to 4.2) in the MQ + AS group (n = 49) (Figure 8A). In study SP-C-005-06, in patients who had gametocytes at baseline, median log_10_ [AUC gametocyte density] was 2.5 (range −0.8 to 4.8) in the PA group (n = 66) and 2.1 (−0.3 to 3.6) in the AL group (n = 22) (Figure 8C). In patients with no gametocytes at baseline, but who had post-baseline gametocytes, in study SP-C-004-06, median log_10_ [AUC gametocyte density] was 2.1 (range 0.43 to 3.65) in the PA group (n = 64) and 1.4 (range 0.22 to 3.4) in the MQ + AS group (n = 17) (Figure 8B). In study SP-C-005-06, median log_10_ [AUC gametocyte density] was 0.9 (range −0.5 to 3.0) in the PA group (n = 67) and 0.5 (−0.5 to 3.2) in the AL group (n = 25) (Figure 8D).

## Discussion

The integrated safety analysis included 2,815 adults and children treated with PA for uncomplicated *P. falciparum* or *P. vivax* infection. The PA group was well balanced for baseline clinical and demographic characteristics with respect to the combined comparator group. The safety profile for PA in this analysis was similar to that reported for pyronaridine and artemisinin monotherapy [21,32-35]. Comparator safety profiles were also consistent with previous reports for MQ + AS [36-42], AL [25,43-46] and CQ [23,47,48].

Overall, PA and comparators were generally well tolerated. Although the nature and incidence of adverse events were generally similar between PA and comparators overall, dizziness was more common with MQ + AS (6.6%) and myalgia with CQ (9.2%) than with PA (1.4% and 3.8%, respectively) (Table 4). Serious adverse events were uncommon in all treatment groups (0–0.7%). For PA, only one patient had serious adverse events thought to be related to drug treatment; hepatic enzymes increased and incomplete abortion.

The only significant laboratory finding in the PA group was a mean increase in ALT and AST on days 3 and 7, compared with decreases in these enzymes for comparators (Figure 2). As a result of this signal, an Independent Data Monitoring Committee (IDMC) comprising six members, including three experts in hepatotoxicity, examined the data from all of the studies. Increased transaminases have been observed consistently across all the Phase II/III PA clinical studies and two (of four) of the completed Phase I studies. When they occurred, rises in transaminases peaked by day 7 and levels had normalized or were decreasing by day 28 with no instances of Grade 3 or 4 toxicity at this time point. Increased transaminases were associated rarely with rises in total bilirubin, which was often high at baseline and subsequently fell during treatment. Overall, 0.2% (7/2,815) of patients in the PA group and 0.3% (2/603) in the AL group had ALT and/or AST >3x the upper limit of normal (ULN) plus peak total bilirubin >2xULN; one of these patients in the PA group had elevated ALP which precluded them as a Hy’s law case. Importantly, no patients had any clinical sequelae related to these liver function changes and as a result the IDMC concluded that whilst PA treatment is associated with transient elevated transaminases, the early onset (day 3–7) and rapid resolution are consistent with a direct low-level toxicity. Therefore, as PA is dosed for only three days, the risk of progressive liver injury is small. Additional studies are ongoing in healthy volunteers and in patients to assess the hepatic safety profile of PA when the treatment is administered more than once.

Electrocardiograph results did not suggest any cardiac safety concerns with PA. As would be expected, prolonged QT interval was uncommon with PA (0.07%) but occurred more frequently with the quinoline derivatives MQ (0.7%) and particularly CQ (2.7%) [49]. Bradycardia was observed as an adverse event in 1.1% of patients receiving PA and 0.8% with AL. However, the finding of bradycardia in a young and otherwise fit population is likely to be associated with the resolution of the tachycardia associated with fever as decreases in mean heart rate were noted across all treatment groups and have been observed in other studies of anti-malarial therapy as patients become afebrile and they return to their normal baseline heart rate [33,50-52].

For the treatment of uncomplicated *P. falciparum* in children and adults, there was no difference between PA and comparators for day-28 PCR-corrected ACPR for the ITT or PP analyses (Figure 3). This reflects results from the three *P. falciparum* Phase III studies in which non-inferiority of PA day 28 PCR-corrected ACPR (PP population) was demonstrated *versus* MQ + AS or AL [15,22,25]. Sub-group analysis showed similar day-28 PCR-corrected ACPR (ITT population) for PA by region, age group, gender, weight, previous malaria, malaria in the last 12 months, and baseline parasitemia *versus* efficacy with PA overall (Table 5).

Kaplan-Meier analysis of the integrated efficacy analysis indicated a *P. falciparum* recrudescence rate for PA intermediate between the comparators (Figure 4A). For individual studies, recrudescence rate over the 42-day study period was marginally higher with PA *versus* MQ + AS (*P* = .049) in SP-C-004-06 and similar *versus* AL (*P* = .90) in SP-C-005-06 and in the paediatric study SP-C-007-07 (*P* = .53) [15,22,25]. Similarly, Kaplan-Meier analysis of the integrated efficacy analysis indicated a *P. falciparum* re-infection rate for PA intermediate between the comparators (AL highest, MQ + AS lowest) (Figure 4B). However, in the individual Phase III studies, re-infection rate was higher with MQ + AS than PA at day 28 (*P* = .04), and similar at day 42 (*P* = .17) [15]. One difference in the integrated analysis was that there were more patients in the PA group who where under 18 years of age than in the MQ + AS group and this may have increased the re-infection rate for PA relative to MQ + AS. In support of this, in the Phase III study of PA *versus* AL that comprised mostly adults (SP-C-005-06), PA had a lower re-infection rate than AL (day 28 *P* = .004, day 42 *P* = .007) [25], but this difference was not evident in the paediatric PA *versus* AL study (SP-C-007-07) (*P* = .77) [22]. In the integrated analysis, re-infection rate was lower with PA than AL and the patients in the AL group tended to be younger. An alternative explanation is that there were differences in transmission rates between the treatment groups given the imbalance in Asian *versus* African centres in the integrated analysis.

In the integrated analysis, the *P. falciparum* parasite clearance rate was 24.1 h with PA and 32.9 h with MQ + AS (Figure 5A, Table 6). However, this was not seen in the comparative Phase III trial of these treatments in which median parasite clearance time was approximately 32 h in both arms (*P* = .08) [15]. This is probably because of the greater proportion of patients from Africa in the PA group *versus* the MQ + AS group in the integrated analysis; median parasite clearance rates were longer in Asia than Africa for all comparators (Table 6). In particular, in Cambodia parasite clearance times were extended for PA and MQ + AS to around 64 h (Figure 5B). However, this did not seem to affect the day-28 PCR-corrected ACPR (ITT population) between Cambodia and Thailand for either PA or MQ + AS. Extended parasite clearance times indicate the presence of artemisinin resistance and the results presented here are consistent with other reports from the Cambodia–Thailand border area [8-14,53]. For example, mean parasite clearance times of around 65 h have been reported for artemisinin-piperaquine, dihydroartemisinin-piperaquine and AL in this region [13].

Parasite clearance times were similar between PA and AL in the integrated analysis (Figure 5A). However, in the individual studies of PA *versus* AL, parasite clearance was faster with PA (*P* = .02) in study SP-C-007-07 conducted in children, and in study SP-C-005-06 including mostly adults (*P* < .001) [22,25]. As above, these differences are probably explained by the longer parasite clearance times in Asian *versus* African centres, there being a relative lack of patients recruited from Asian centres in the AL group compared with the PA group in the integrated analysis.

Fever clearance time for PA in the integrated analysis was approximately intermediate between AL (fastest) MQ + AS (slowest) (Figure 6). However, in the individual Phase III studies, fever clearance times were shorter with PA *versus* AL in the paediatric study (*P* = .049) and there was no difference in the study conducted *versus* AL mostly in adults (*P* = .55) [22,25]. This again probably reflects the greater proportion of patients recruited from Asian centres in the PA and MQ + AS groups *versus* the AL group in the integrated analysis.

In the integrated analysis, it appeared that AL more rapidly eradicated gametocytes *versus* PA and MQ + AS (Figure 7). However, this is also probably explained by the difference in baseline populations. To investigate further, gametocytes carriage was analysed in the two studies that enrolled both adults and children [15,25], using log_10_ AUC [gametocyte density] [54,55]. In patients with baseline gametocytes, MQ + AS did appear to more rapidly reduce gametocyte carriage *versus* PA, though in patients with no gametocytes at baseline PA appeared to more effectively suppress gametocyte emergence (Figure 8). There appeared to be no differences between AL and PA in respect to gametocyte clearance or suppression.

Children are the most important target group for anti-malarial therapy, having reduced immunity and consequently poorer outcomes. Despite this, until recently paediatric ACT formulations have been generally lacking [56]. Paediatric formulations are easier to administer and may be better tolerated, particularly in terms of vomiting, which may affect anti-malarial drug levels [57]. A granule formulation was included in the PA development plan to ensure early availability after drug registration. For the primary efficacy outcome used in the Phase III studies – day-28 PCR-corrected ACPR – the granule and tablet formulation had similar efficacy in this analysis. Efficacy outcomes at day 42 were also similar for the two pyronaridine-artesunate formulations.

For the efficacy analysis, this report is concerned with the integrated analysis of the PA Phase III *P. falciparum* clinical trials. However, the efficacy of PA in *P. vivax* has also been evaluated in one Phase III study in adults and children [23]. In summary, for the primary outcome – the day-14 crude cure rate (PP population) – PA efficacy was 99.5%, (217/218; 95% CI 97.5, 100). This was non-inferior to CQ 100% efficacy (209/209; 95% CI 98.3, 100); treatment difference −0.5% (95% CI −2.6, 1.4). Non-inferiority of PA to CQ was maintained throughout follow-up (days 21, 28, 35 and 42) [23]. Additional clinical trials will report PA safety and efficacy in young children with *P. vivax* malaria and PA efficacy in areas of chloroquine-resistant *P. vivax*.

## Conclusions

Overall, PA was well tolerated with a similar adverse event profile to comparators. Although PA was associated with transient increases in transaminases in a relatively small proportion of patients, there was no indication of a risk of progressive liver injury. Pyronaridine-artesunate efficacy against *P. falciparum* was consistently high regardless of geographical region, patient age, gender, or degree of parasitaemia. Against *P. vivax*, PA had efficacy at least as good as CQ but with more rapid parasite and fever clearance and a lower parasite re-emergence rate. Importantly, PA has been developed to have a specific paediatric granule formulation available, shown to have equivalent pharmacokinetics and efficacy to the tablet formulation [24]. Initially, PA will be used in areas of low endemicity and where cases of artemisinin resistance have been reported with deployment to areas of high endemicity once further data on repeated treatment with PA have been obtained. Pyronaridine-artesunate is a useful new ACT and should be a valuable addition to anti-malarial treatment programmes.

## Competing interest

SD, and IB-F are employees of MMV; JCC is a former employee of MMV; SA-B and RMM are contractors employed by MMV; C-SS is a former employee of Shin Poong Pharmaceutical Co. Ltd.; LF is an advisor to MMV.

## Authors’ contributions

All authors were involved in study design and conduct, contributed to the paper and approved the final version for submission.

## References

[B1] World Health Organization World Malaria Report 2011 http://www.who.int/malaria/world_malaria_report_2011/9789241564403_eng.pdf

[B2] Roll Back Malaria Partnership Secretariat World Malaria Day 2010 *Africa Update* http://www.rollbackmalaria.org/ProgressImpactSeries/docs/wmd2010report-en.pdf

[B3] Mueller I Galinski MR Baird JK Carlton JM Kochar DK Alonso PL del Portillo HA Key gaps in the knowledge of *Plasmodium vivax*, a neglected human malaria parasite Lancet Infect Dis 2009 9 555 566 10.1016/S1473-3099(09)70177-X 19695492

[B4] Price RN Tjitra E Guerra CA Yeung S White NJ Anstey NM Vivax malaria: neglected and not benign Am J Trop Med Hyg 2007 77 79 87 18165478 PMC2653940

[B5] Guerra CA Snow RW Hay SI Mapping the global extent of malaria in 2005 Trends Parasitol 2006 22 353 358 10.1016/j.pt.2006.06.006 16798089 PMC3111076

[B6] Guerra CA Howes RE Patil AP Gething PW Van Boeckel TP Temperley WH Kabaria CW Tatem AJ Manh BH Elyazar IR Baird JK Snow RW Hay SI The international limits and population at risk of *Plasmodium vivax* transmission in 2009 PLoS Negl Trop Dis 2009 4 e774 20689816 10.1371/journal.pntd.0000774 PMC2914753

[B7] World Health Organization Guidelines for the treatment of malaria (second edition) http://whqlibdoc.who.int/publications/2010/9789241547925_eng.pdf

[B8] Wongsrichanalai C Wimonwattrawatee T Sookto P Laoboonchai A Heppner DG Kyle DE Wernsdorfer WH *In vitro* sensitivity of *Plasmodium falciparum* to artesunate in Thailand Bull World Health Organ 1999 77 392 398 10361756 PMC2557670

[B9] Noedl H Se Y Schaecher K Smith BL Socheat D Fukuda MM Evidence of artemisinin-resistant malaria in western Cambodia N Engl J Med 2008 359 2619 2620 10.1056/NEJMc0805011 19064625

[B10] World Health Organization Containment of malaria multi-drug resistance on the Cambodia-Thailand border Report of an informal consultation, Phnom Penh *29–30 January 2007* http://www.who.int/malaria/publications/multi_drug_resistance_en.pdf

[B11] World Health Organization Global malaria control and elimination: report of a meeting on containment of artemisinin tolerancehttp://whqlibdoc.who.int/publications/2008/9789241596817_eng.pdf

[B12] Dondorp AM Nosten F Yi P Das D Phyo AP Tarning J Lwin KM Ariey F Hanpithakpong W Lee SJ Ringwald P Silamut K Imwong M Chotivanich K Lim P Herdman T An SS Yeung S Singhasivanon P Day NP Lindegardh N Socheat D White NJ Artemisinin resistance in *Plasmodium falciparum* malaria N Engl J Med 2009 361 455 467 10.1056/NEJMoa0808859 19641202 PMC3495232

[B13] Song J Socheat D Tan B Seila S Xu Y Ou F Sokunthea S Sophorn L Zhou C Deng C Wang Q Li G Randomized trials of artemisinin-piperaquine, dihydroartemisinin-piperaquine phosphate and artemether-lumefantrine for the treatment of multi-drug resistant falciparum malaria in Cambodia-Thailand border area Malar J 2011 10 231 10.1186/1475-2875-10-231 21827706 PMC3169515

[B14] Wongsrichanalai C Meshnick SR Declining artesunate-mefloquine efficacy against falciparum malaria on the Cambodia-Thailand border Emerg Infect Dis 2008 14 716 719 10.3201/eid1405.071601 18439351 PMC2600243

[B15] Rueangweerayut R Phyo A Uthaisin C Poravuth Y Binh T Tinto H Pénali L Valecha N Tien N Abdulla S Borghini-Fuhrer I Duparc S Shin C-S Fleckenstein L Pyronaridine-artesunate *versus* mefloquine plus artesunate for malaria NEJM 2012 366 1298 1309 10.1056/NEJMoa1007125 22475593

[B16] Phyo AP Nkhoma S Stepniewska K Ashley EA Nair S McGready R Ler Moo C Al-Saai S Dondorp AM Lwin KM Singhasivanon P Day NP White NJ Anderson TJ Nosten F Emergence of artemisinin-resistant malaria on the western border of Thailand: a longitudinal study Lancet 2012 379 1960 1966 10.1016/S0140-6736(12)60484-X 22484134 PMC3525980

[B17] Douglas NM Anstey NM Angus BJ Nosten F Price RN Artemisinin combination therapy for vivax malaria Lancet Infect Dis 2010 10 405 416 10.1016/S1473-3099(10)70079-7 20510281 PMC3350863

[B18] Baird JK Resistance to therapies for infection by *Plasmodium vivax* Clin Microbiol Rev 2009 22 508 534 10.1128/CMR.00008-09 19597012 PMC2708388

[B19] Mueller I Widmer S Michel D Maraga S McNamara DT Kiniboro B Sie A Smith TA Zimmerman PA High sensitivity detection of *Plasmodium* species reveals positive correlations between infections of different species, shifts in age distribution and reduced local variation in Papua New Guinea Malar J 2009 8 41 10.1186/1475-2875-8-41 19284594 PMC2657150

[B20] World Health Organization Regional Office for the Western Pacific Interregional workshop on the control of vivax malaria in East Asia (Shanghai, China, 17–20 November 2003) http://whqlibdoc.who.int/wpro/2004/RS_2003_GE_42(CHN).pdf

[B21] Croft SL Duparc S Arbe-Barnes SJ Craft JC Shin CS Fleckenstein L Borghini-Fuhrer I Rim HJ Review of pyronaridine anti-malarial properties and product characteristics Malar J 2012 11 270 10.1186/1475-2875-11-270 22877082 PMC3483207

[B22] Kayentao K Doumbo OK Pénali LK Offianan AT Bhatt KM Kimani J Tshefu AK Tambwe JKH Ramharter M Martinez de Salazar P Tiono AB Ouédraogo A Bustos MDG Quicho F Borghini-Fuhrer I Duparc s Shin C-S Fleckenstein L Pyronaridine-artesunate granules *versus* artemether-lumefantrine crushed tablets in children with Plasmodium falciparum malaria: a randomized controlled trial Malar J 2012 11 364 10.1186/1475-2875-11-364 23113947 PMC3566922

[B23] Poravuth Y Socheat D Rueangweerayut R Uthaisin C Pyae Phyo A Valecha N Rao BH Tjitra E Purnama A Borghini-Fuhrer I Duparc S Shin CS CS Fleckenstein L Pyronaridine-artesunate *versus* chloroquine in patients with acute *Plasmodium vivax* malaria: a randomized, double-blind, non-inferiority trial PLoS One 2011 6 e14501 10.1371/journal.pone.0014501 21267072 PMC3022577

[B24] Ramharter M Kurth F Schreier AC Nemeth J Glasenapp I Belard S Schlie M Kammer J Koumba PK Cisse B Mordmuller B Lell B Issifou S Oeuvray C Fleckenstein L Kremsner PG Fixed-dose pyronaridine-artesunate combination for treatment of uncomplicated falciparum malaria in pediatric patients in Gabon J Infect Dis 2008 198 911 919 10.1086/591096 18694333

[B25] Tshefu AK Gaye O Kayentao K Thompson R Bhatt KM Sesay SS Bustos DG Tjitra E Bedu-Addo G Borghini-Fuhrer I Duparc S Shin CS Fleckenstein L Efficacy and safety of a fixed-dose oral combination of pyronaridine-artesunate compared with artemether-lumefantrine in children and adults with uncomplicated *Plasmodium falciparum* malaria: a randomised non-inferiority trial Lancet 2010 375 1457 1467 10.1016/S0140-6736(10)60322-4 20417857

[B26] Price RN Marfurt J Chalfein F Kenangalem E Piera KA Tjitra E Anstey NM Russell B *In vitro* activity of pyronaridine against multidrug-resistant *Plasmodium falciparum* and *Plasmodium vivax* Antimicrob Agents Chemother 2010 54 5146 5150 10.1128/AAC.00801-10 20876370 PMC2981240

[B27] Okombo J Kiara SM Mwai L Pole L Ohuma E Ochola LI Nzila A Baseline *in vitro* activities of the antimalarials pyronaridine and methylene blue against *Plasmodium falciparum* isolates from Kenya Antimicrob Agents Chemother 2012 56 1105 1107 10.1128/AAC.05454-11 22123687 PMC3264213

[B28] Zhang CL Zhou HN Wang J Liu H [*In vitro* sensitivity of *Plasmodium falciparum* isolates from China-Myanmar border region to chloroquine, piperaquine and pyronaridine] Zhongguo Ji Sheng Chong Xue Yu Ji Sheng Chong Bing Za Zhi 2012 30 41 44 22913189

[B29] World Health Organization Assessment and monitoring of antimalarial drug efficacy for the treatment of uncomplicated falciparum malaria (WHO/HTM/RBM/2003.50) http://whqlibdoc.who.int/hq/2003/WHO_HTM_RBM_2003.50.pdf

[B30] Felger I Beck HP Genotyping of *Plasmodium falciparum.* PCR-RFLP analysis Methods Mol Med 2002 72 117 129 12125107 10.1385/1-59259-271-6:117

[B31] Medicines for Malaria Venture, World Health Organization Methods and techniques for clinical trials on antimalarial drug efficacy: genotyping to identify parasite populationshttp://whqlibdoc.who.int/publications/2008/9789241596305_eng.pdf

[B32] Price R van Vugt M Phaipun L Luxemburger C Simpson J McGready R ter Kuile F Kham A Chongsuphajaisiddhi T White NJ Nosten F Adverse effects in patients with acute falciparum malaria treated with artemisinin derivatives Am J Trop Med Hyg 1999 60 547 555 10348227 10.4269/ajtmh.1999.60.547

[B33] Ribeiro IR Olliaro P Safety of artemisinin and its derivatives. A review of published and unpublished clinical trials Med Trop (Mars) 1998 58 50 53 10212898

[B34] Ringwald P Bickii J Basco L Randomised trial of pyronaridine *versus* chloroquine for acute uncomplicated falciparum malaria in Africa Lancet 1996 347 24 28 10.1016/S0140-6736(96)91558-5 8531545

[B35] Ringwald P Bickii J Basco LK Efficacy of oral pyronaridine for the treatment of acute uncomplicated falciparum malaria in African children Clin Infect Dis 1998 26 946 953 10.1086/513942 9564481

[B36] Agomo PU Meremikwu MM Watila IM Omalu IJ Odey FA Oguche S Ezeiru VI Aina OO Efficacy, safety and tolerability of artesunate-mefloquine in the treatment of uncomplicated *Plasmodium falciparum* malaria in four geographic zones of Nigeria Malar J 2008 7 172 10.1186/1475-2875-7-172 18782445 PMC2542389

[B37] Bhatt KM Samia BM Bhatt SM Wasunna KM Efficacy and safety of an artesunate/mefloquine combination, (artequin) in the treatment of uncomplicated *P. falciparum* malaria in Kenya East Afr Med J 2006 83 236 242 16866217 10.4314/eamj.v83i5.9428

[B38] Bouyou-Akotet MK Ramharter M Ngoungou EB Mamfoumbi MM Mihindou MP Missinou MA Kurth F Belard S Agnandji ST Issifou S Heidecker JL Trapp S Kremsner PG Kombila M Efficacy and safety of a new pediatric artesunate-mefloquine drug formulation for the treatment of uncomplicated falciparum malaria in Gabon Wien Klin Wochenschr 2010 122 173 178 10.1007/s00508-010-1317-1 20361381

[B39] Frey SG Chelo D Kinkela MN Djoukoue F Tietche F Hatz C Weber P Artesunate-mefloquine combination therapy in acute *Plasmodium falciparum* malaria in young children: a field study regarding neurological and neuropsychiatric safety Malar J 2010 9 291 10.1186/1475-2875-9-291 20964849 PMC2984569

[B40] Krudsood S Looareesuwan S Tangpukdee N Wilairatana P Phumratanaprapin W Leowattana W Chalermrut K Ramanathan S Navaratnam V Olliaro P Vaillant M Kiechel JR Taylor WR New fixed-dose artesunate-mefloquine formulation against multidrug-resistant *Plasmodium falciparum* in adults: a comparative phase IIb safety and pharmacokinetic study with standard-dose nonfixed artesunate plus mefloquine Antimicrob Agents Chemother 2010 54 3730 3737 10.1128/AAC.01187-09 20547795 PMC2935027

[B41] Massougbodji A Kone M Kinde-Gazard D Same-Ekobo A Cambon N Mueller EA A randomized, double-blind study on the efficacy and safety of a practical three-day regimen with artesunate and mefloquine for the treatment of uncomplicated *Plasmodium falciparum* malaria in Africa Trans R Soc Trop Med Hyg 2002 96 655 659 10.1016/S0035-9203(02)90344-5 12625145

[B42] Mayxay M Keomany S Khanthavong M Souvannasing P Stepniewska K Khomthilath T Keola S Pongvongsa T Phompida S Ubben D Valecha N White NJ Newton PN A phase III, randomized, non-inferiority trial to assess the efficacy and safety of dihydroartemisinin-piperaquine in comparison with artesunate-mefloquine in patients with uncomplicated *Plasmodium falciparum* malaria in southern Laos Am J Trop Med Hyg 2010 83 1221 1229 10.4269/ajtmh.2010.10-0276 21118925 PMC2990035

[B43] Abdulla S Sagara I Borrmann S D’Alessandro U Gonzalez R Hamel M Ogutu B Martensson A Lyimo J Maiga H Sasi P Nahum A Bassat Q Juma E Otieno L Bjorkman A Beck HP Andriano K Cousin M Lefevre G Ubben D Premji Z Efficacy and safety of artemether-lumefantrine dispersible tablets compared with crushed commercial tablets in African infants and children with uncomplicated malaria: a randomised, single-blind, multicentre trial Lancet 2008 372 1819 1827 10.1016/S0140-6736(08)61492-0 18926569

[B44] Hatz C Soto J Nothdurft HD Zoller T Weitzel T Loutan L Bricaire F Gay F Burchard GD Andriano K Lefevre G De Palacios PI Genton B Treatment of acute uncomplicated falciparum malaria with artemether-lumefantrine in nonimmune populations: a safety, efficacy, and pharmacokinetic study Am J Trop Med Hyg 2008 78 241 247 18256423

[B45] Mueller EA van Vugt M Kirch W Andriano K Hunt P de Palacios PI Efficacy and safety of the six-dose regimen of artemether-lumefantrine for treatment of uncomplicated *Plasmodium falciparum* malaria in adolescents and adults: a pooled analysis of individual patient data from randomized clinical trials Acta Trop 2006 100 41 53 10.1016/j.actatropica.2006.09.007 17045558

[B46] Sagara I Rulisa S Mbacham W Adam I Sissoko K Maiga H Traore OB Dara N Dicko YT Dicko A Djimde A Jansen FH Doumbo OK Efficacy and safety of a fixed dose artesunate-sulphamethoxypyrazine-pyrimethamine compared to artemether-lumefantrine for the treatment of uncomplicated falciparum malaria across Africa: a randomized multi-centre trial Malar J 2009 8 63 10.1186/1475-2875-8-63 19366448 PMC2678145

[B47] Naing C Aung K Win DK Wah MJ Efficacy and safety of chloroquine for treatment in patients with uncomplicated *Plasmodium vivax* infections in endemic countries Trans R Soc Trop Med Hyg 2010 104 695 705 10.1016/j.trstmh.2010.08.009 20850161

[B48] Mengesha T Makonnen E Comparative efficacy and safety of chloroquine and alternative antimalarial drugs: a meta-analysis from six African countries East Afr Med J 1999 76 314 319 10750517

[B49] White NJ Cardiotoxicity of antimalarial drugs Lancet Infect Dis 2007 7 549 558 10.1016/S1473-3099(07)70187-1 17646028

[B50] Karbwang J Na-Bangchang K Thanavibul A Bunnag D Chongsuphajaisiddhi T Harinasuta T Comparison of oral artesunate and quinine plus tetracycline in acute uncomplicated falciparum malaria Bull World Health Organ 1994 72 233 238 8205643 PMC2486535

[B51] Karbwang J Bangchang KN Thanavibul A Bunnag D Chongsuphajaisiddhi T Harinasuta T Comparison of oral artemether and mefloquine in acute uncomplicated falciparum malaria Lancet 1992 340 1245 1248 10.1016/0140-6736(92)92947-E 1359318

[B52] Sowunmi A Oduola AM Efficacy of artemether in severe falciparum malaria in African children Acta Trop 1996 61 57 63 10.1016/0001-706X(95)00143-3 9133165

[B53] Jambou R Legrand E Niang M Khim N Lim P Volney B Ekala MT Bouchier C Esterre P Fandeur T Mercereau-Puijalon O Resistance of *Plasmodium falciparum* field isolates to in-vitro artemether and point mutations of the SERCA-type PfATPase6 Lancet 2005 366 1960 1963 10.1016/S0140-6736(05)67787-2 16325698

[B54] Bousema T Okell L Shekalaghe S Griffin JT Omar S Sawa P Sutherland C Sauerwein R Ghani AC Drakeley C Revisiting the circulation time of *Plasmodium falciparum* gametocytes: molecular detection methods to estimate the duration of gametocyte carriage and the effect of gametocytocidal drugs Malar J 2010 9 136 10.1186/1475-2875-9-136 20497536 PMC2881938

[B55] Sowunmi A Balogun T Gbotosho GO Happi CT Adedeji AA Bolaji OM Fehintola FA Folarin OA Activities of artemether-lumefantrine and amodiaquine-sulfalene-pyrimethamine against sexual-stage parasites in falciparum malaria in children Chemotherapy 2008 54 201 208 10.1159/000140463 18560227

[B56] Agnandji ST Kurth F Belard S Mombo-Ngoma G Basra A Fernandes JF Soulanoudjingar SS Adegnika AA Ramharter M Current status of the clinical development and implementation of paediatric artemisinin combination therapies in Sub-Saharan Africa Wien Klin Wochenschr 2011 123 1 7 9 10.1007/s00508-011-0039-3 21826416

[B57] Kurth F Belard S Adegnika AA Gaye O Kremsner PG Ramharter M Do paediatric drug formulations of artemisinin combination therapies improve the treatment of children with malaria? A systematic review and meta-analysis Lancet Infect Dis 2010 10 125 132 10.1016/S1473-3099(09)70327-5 20113981

